# Learning hyperparameter predictors for similarity-based multidisciplinary topology optimization

**DOI:** 10.1038/s41598-023-42009-0

**Published:** 2023-09-08

**Authors:** Mariusz Bujny, Muhammad Salman Yousaf, Nathan Zurbrugg, Duane Detwiler, Stefan Menzel, Satchit Ramnath, Thiago Rios, Fabian Duddeck

**Affiliations:** 1 Honda Research Institute Europe GmbH, 63073 Offenbach, Germany; 2 Technical University of Munich, Munich, 80333 Germany; 3 Honda Development and Manufacturing of America, LLC, Raymond, OH 43067 USA; 4 Honda Research Institute USA, Inc., Columbus, OH 43212 USA; 5 The Ohio State University, Columbus, OH 43210 USA

**Keywords:** Mechanical engineering, Computational science

## Abstract

Topology optimization (TO) plays a significant role in industry by providing engineers with optimal material distributions based exclusively on the information about the design space and loading conditions. Such approaches are especially important for current multidisciplinary design tasks in industry, where the conflicting criteria often lead to very unintuitive solutions. Despite the progress in integrating manufacturing constraints into TO, one of the main factors restricting the use of TO in practice is the users’ limited control of the final material distribution. To address this problem, recently, a universal methodology for enforcing similarity to reference structures in various TO methods by applying scaling of elemental energies was proposed. The method, however, requires an expensive hyperparameter sampling, which involves running multiple TO processes to find the design of a given similarity to a reference structure. In this article, we propose a novel end-to-end approach for similarity-based TO, which integrates a machine learning model to predict the hyperparameters of the method, and provide the engineer, at minimal computational cost, with a design satisfying multidisciplinary criteria expressed by the similarity to a reference. The training set for the model is generated based on an academic linear elastic problem, but the model generalizes well to both nonlinear dynamic crash and industrial-scale TO problems. We show the latter by applying the proposed methodology to a real-world multidisciplinary TO problem of a car hood frame, which demonstrates the usefulness of the approach in industrial settings.

## Introduction

Structural topology optimization (TO) methods are computational techniques which optimize the distribution of a given material within a specified design space based on boundary and initial conditions, e.g., supports, forces, or impact velocities in case of dynamic problems. The high-dimensional design representations used in TO, where often every finite element of the simulation model is parametrized by an individual design variable, give the computer program the highest level of flexibility, resulting in complex structural concepts of superior performance. At the same time, the multidisciplinary character of the problems encountered in engineering practice nowadays makes the traditional trial-and-error design process more and more challenging due to very unintuitive solutions designers have to come up with to fulfill stringent performance, environmental, and economic requirements. In such a case, utilization of numerical optimization methods such as structural TO seems a natural choice, allowing for an efficient mitigation of limited human capabilities to manage problems involving multitudes of conflicting design criteria.

Traditionally, structural TO methods are divided into density-based approaches, including homogenization methods^1^ as well as Solid Isotropic Material with Penalization (SIMP) based techniques^2^, and Level Set Methods (LSMs)^3^. More recently, also feature-mapping techniques^4^, e.g., approaches using Moving Morphable Components (MMCs)^5^ as a design representation, gained a lot of attention thanks to the decoupling of the design parametrization from the discretized model used for simulation, which allows for a more straightforward integration of manufacturing limitations^6,7^, and, through reduction of the dimensionality of the optimization problem, enables efficient use of even nongradient techniques to address highly nonlinear dynamic problems^8–12^. At the same time, also heuristic, nongradient TO approaches such as Hybrid Cellular Automata (HCA)^13,14^ or Bi-directional Evolutionary Structural Optimization (BESO)^15^ are increasingly used to address complex multidisciplinary problems, which is possible due to their general character. Thanks to such research developments, it becomes feasible to incorporate simulation models of various physical phenomena into TO and go beyond linear elastic static problems addressed in standard approaches, which answers very well the needs of the industry.

Apart from well-established methods for multidisciplinary sizing and shape optimization^16–19^, the TO approaches involving multiple design criteria and different physics are under continuous development. In the context of the automotive industry, especially the methods allowing for a concurrent optimization considering static and crash, as well as Noise, Vibration, and Harshness (NVH) load cases^20–23^ play an important role in defining structural concepts in the early phases of development. Moreover, in practical applications, considering economic factors associated with manufacturing technology is critical and was also addressed in the TO research in the recent years^24–26^. Nevertheless, there are still many design requirements which are virtually impossible to describe in a closed mathematical form. Usually, due to the economic limitations of the manufacturing and assembly process, it is much more efficient to re-use components developed in the previous product design cycles and increase the commonality among different models. For instance, in the automotive industry, often a common platform, i.e., a set of components shared among different models, is developed to maximize the cost efficiency of the production process. Moreover, during the re-integration of a topologically-optimized design into a larger system, it is required to add features whose requirements were difficult to account for in the optimization, but play an important role in the assembly process. Typically, standard TO approaches eliminate such features. Finally, in cases where aesthetics are important, such as rim design^27^, it is not unusual to strive for novelty in the design process in order to explore new structural concepts, which can be accomplished, e.g., by maximizing the dissimilarity to an entire set of known designs^27,28^. Often, enhancing design space exploration by enforcing generation of diverse solutions is also important in the context of improving global search capabilities of nongradient optimizers^29–31^. Considering these additional optimization criteria, related often to subjective preferences of a designer and a specific know-how of the company, which can be seen as an entire range of disciplines not related to any physical load cases, it is extremely difficult to utilize TO to directly derive practically useful design concepts, and alternative methods are needed to address this problem.

**Figure 1:**
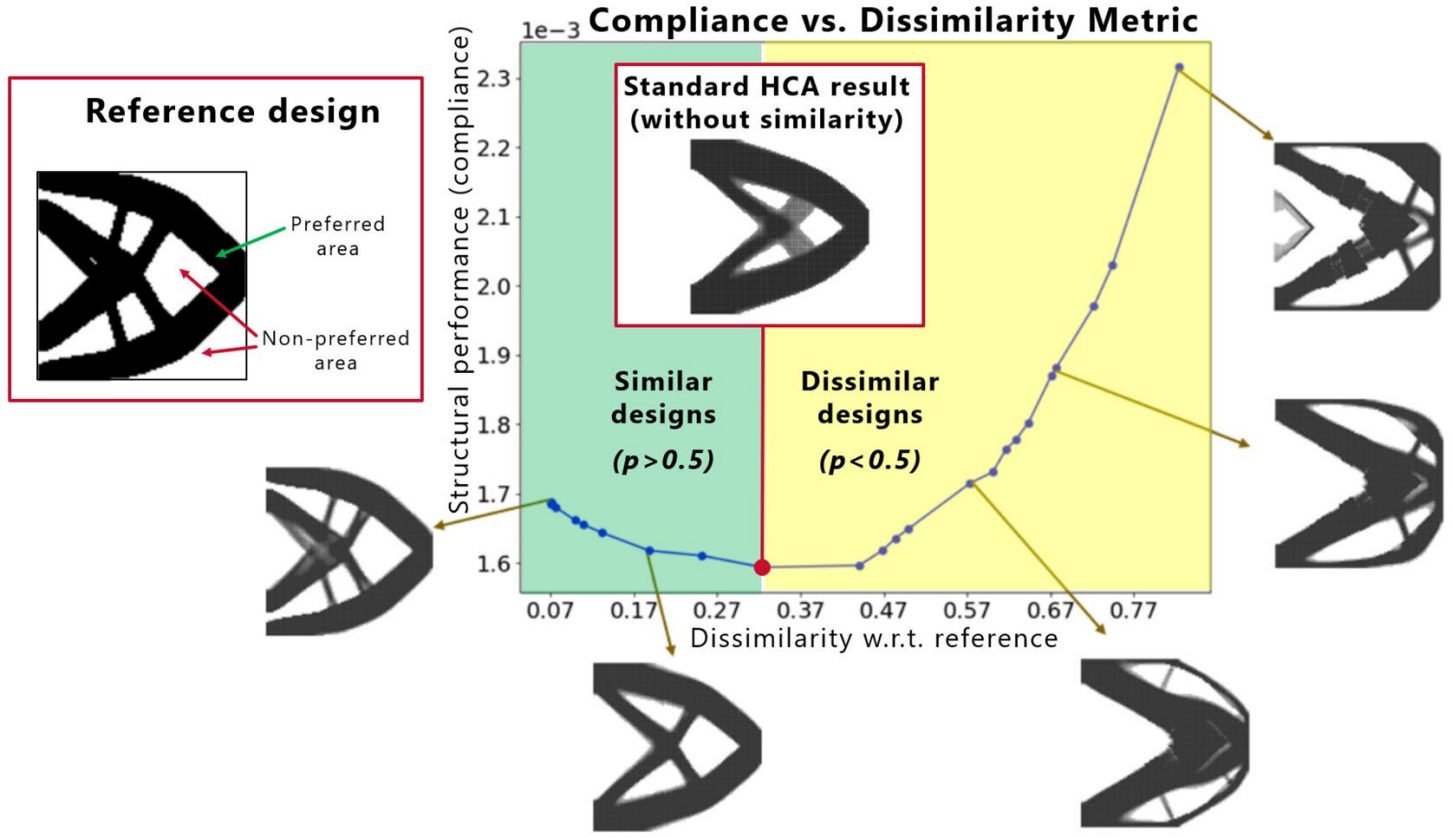
An example of similarity-based TO using ESM in HCA. The reference design used for computation of the dissimilarity metric *s* is shown in the red box on the left hand side. Each of the points on the blue curve corresponds to a TO run for a different value of the energy scaling factor *p*. Designs similar to the reference are obtained for $p>0.5$, while the dissimilar ones are generated for $p<0.5$. A similarity-based TO for $p=0.5$ yields the same design as a standard TO without similarity constraints.

A very promising methodology able to account for both different structural criteria and nonstructural disciplines mentioned above is TO considering similarity constraints. In one of the first approaches employing this concept, Oh et al.^27^ used gradient-based SIMP together and a Generative Adversarial Network (GAN) for design exploration with an additional design novelty objective, interpreted as geometric dissimilarity. This methodology, however, requires an expensive generation of large amounts of design concepts using TO in order to train the GAN. Moreover, it relies on a gradient-based TO process, which restricts the applicability of the approach to the standard optimization tasks where the gradient information can be derived analytically. In the context of crash load cases, which are essential in the development of car body structures, analytical sensitivities are usually not available^32^ and alternative methods, such as heuristic HCA approach^13,14^ or the MMC-based nongradient techniques^8–11^ have to be used. To address such problems, recently, an Energy Scaling Method (ESM)^33,34^, which is able to control similarity of the structure being optimized to a reference design, was proposed. Since the method directly scales the elemental energies obtained from the finite element simulation model, it is independent of the density update rules utilized in the underlying TO approach, and, therefore, can be used with both gradient-based and nongradient methods like HCA. Figure 1 shows structures of different similarity levels w.r.t. the reference structure obtained using ESM in HCA. By varying the energy scaling factor, a hyperparameter of ESM, and running multiple TO runs, one can obtain an entire spectrum of structures—from the most similar to the reference, through neutral, to the most dissimilar designs. We strongly believe that ESM offers an intuitive and straightforward way of dealing with multiobjective, multidisciplinary problems by efficiently coupling the standard TO approaches using structural optimization criteria with a similarity objective, which can encapsulate requirements from many different disciplines without the need to provide their precise mathematical description, which is often very difficult in case of real-world scenarios.

In recent years, Machine Learning (ML) started to play an increasingly important role in enhancing TO methods. Apart from the approaches aiming for a direct prediction of the optimal design^35–37^ or learning potentially more efficient material parameterization for TO via unsupervised learning^27,38^, also methods taking advantage of lower-dimensional TO representations, e.g., MMCs, together with meta-models of structural responses have been proposed^11,12,39^. In fact, these methodologies belong to a broader category of approaches based on surrogate modelling, which have been successfully used for years in various, industrial-scale design optimization problems involving computationally costly simulations^16,29,40–43^. In more recent approaches, instead of predicting the structural responses based on input features being the design variables, the performance of the structure resulting from the entire TO run can be inferred by providing an ML model with the optimization hyperparameters, e.g., weights of objectives in the aggregated cost function^31^. Finally, alternative use cases of ML in TO involve supervised learning of sensitivities^44,45^ or building models predicting favorable topological variations^46^, as well.

In our previous works^33,34^, we have validated the ESM using standard TO benchmark test cases and demonstrated its usefulness in solving nonlinear dynamic crash as well as industrial-scale problems. Despite the high practical value of ESM, it remained computationally costly, since, to find a design of a given similarity w.r.t. the reference structure, one has to carry out multiple optimization runs for different values of the scaling factor. In this work, to address this problem, we propose a holistic end-to-end similarity-based TO approach capable of handling complex multidisciplinary problems, where an ML model predicts the relationship between the attainable similarity levels and the hyperparameter values of ESM based exclusively on a single, standard TO run. We refer to the framework as an *end-to-end* method since it is able to automatically determine the required hyperparameter value to reach a specific level of similarity. Using the learned hyperparameter predictor, the designer can directly understand the influence of the hyperparameters of ESM on the TO process and specify a priori the required similarity level of the resulting, topologically-optimized design. Despite training of the ML model on a dataset of structures generated using an academic, linear elastic compliance minimization TO problem, the model generalizes well to both nonlinear crash test cases and industrial optimization tasks, which we demonstrate in this work. Moreover, in multiple sensitivity studies, we investigate the properties of the obtained ML model to understand the main factors influencing the similarity-based optimization process.

The remainder of this paper is structured as follows. First, we introduce the novel end-to-end similarity-based TO framework leveraging the potential of ML to automatically predict energy scaling factors. Secondly, based on an ML model’s sensitivity analysis we investigate the influence of relevant design features on the attainable similarity levels. Finally, we apply the end-to-end approach to challenging real-world applications involving nonlinear crash TO as well as 3D large-scale TO of a car hood frame. At the end, we summarize the paper and give an outlook for promising future research directions.

## End-to-end similarity-based topology optimization method for multidisciplinary problems

**Figure 2:**
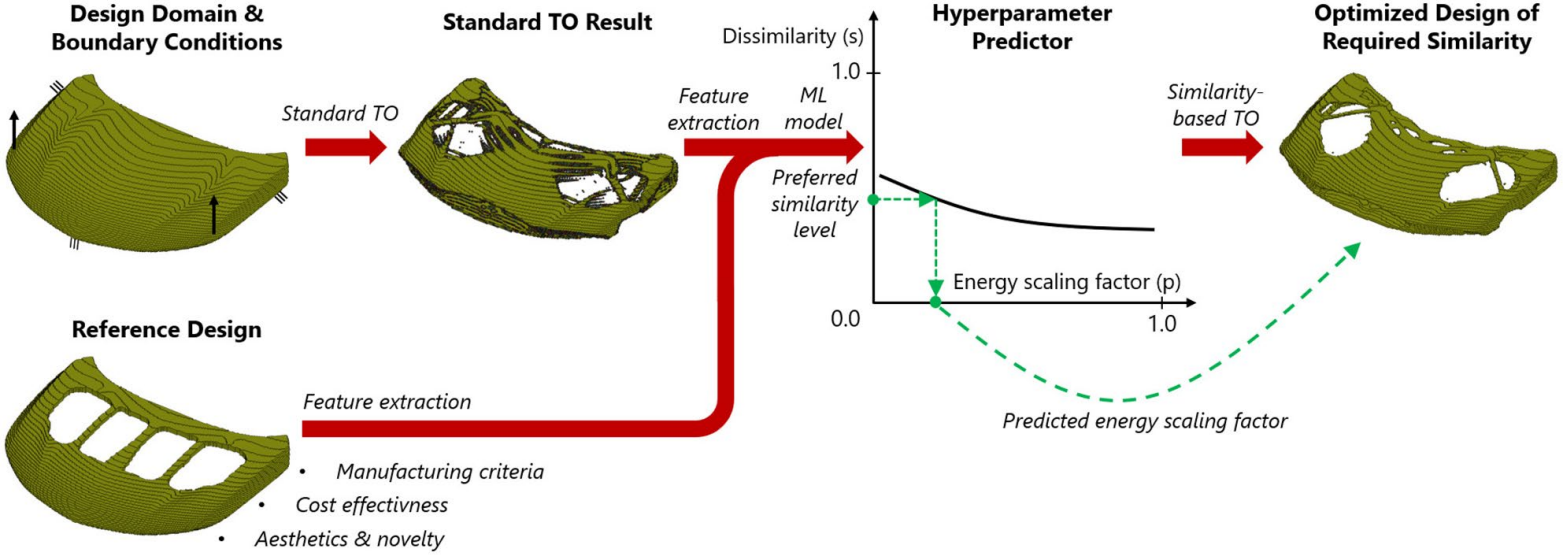
Illustration of the end-to-end framework for multidisciplinary, similarity-based TO using ESM.

This section introduces the holistic, end-to-end TO framework for similarity-driven TO based on ESM. Figure 2 illustrates the flow of information between the main components of the proposed algorithm. As in the standard TO, the user provides the system with a design domain and boundary conditions along with the definition of the optimization problem. Based on this information, a single, standard TO run is performed. In addition, a reference design, which encapsulates the criteria related to multiple alternative disciplines, is defined. The information from the standard TO result and the reference is utilized to extract features which are used by the hyperparameter predictor to determine the relationship between the dissimilarity metric and the energy scaling factor. Depending on a particular dissimilarity metric which is used, the difference between designs can be quantified in various ways. The metric used in this work, which is described in the next section, can be interpreted as a fraction of the design volume where the optimized structure and the reference design are different. Thanks to this intuitive interpretation, the designer can specify the desired volume difference prior to the similarity-based optimization and find an appropriate scaling factor value based on the relationship generated by the hyperparameter predictor. Moreover, by predicting the entire curve, the sensitivity of the dissimilarity metric w.r.t. different scaling factor values can be easily analyzed. Finally, the predicted scaling factor value is used to derive the optimized design of a given similarity level via ESM-based TO.

### Energy scaling method

Following the other methods for similarity-based TO^27,33^, ESM utilizes a dissimilarity metric *s* of the form:

1 $$\begin{aligned} s = \frac{\sum \limits _{e=1}^{N} \left( {x_e}-{x^{ref}_e}\right) ^2}{N}, \end{aligned}$$

with *N*, $x_e$, and $x_e^{ref}$ being the total number of elements in the design domain (for both the design being optimized and the reference structure), relative density of the element *e* in the design subject to optimization, and the relative density of the element *e* in the reference structure, respectively. Please note that in density-based TO, relative densities are the design variables taking values in the range of [0, 1], which influence the stiffness of the elements according to the specific relationship^47^. For instance, in SIMP, the relationship between element density and its Young’s modulus is modeled as a power law in order to penalize the elements with intermediate densities, i.e., $x_e\in (0,1)$. As mentioned before, the metric (1) corresponds approximately to the ratio of the volume (or mass) of the difference between the design being optimized and the reference structure to the total volume (or mass) of the design domain. The approximate character of the metric results from the utilization of continous design variables, however, since both SIMP and HCA try develop designs with densities equal exclusively to 0 or 1, the metric calculated for the final design should be very close to the fraction of the design volume where the optimized structure and the reference design are different.

As indicated in our prior publications^33,34^, ESM was inspired by our observation that a formal integration of the sensitivity of the dissimilarity metric (1) into a gradient-based optimization algorithm, e.g., Optimality Criteria method^47^, for solving a compliance minimization problem, leads to an update rule which scales up the elemental energies in the areas occupied by the reference structure and scales down the energies of all the other elements. Intuitively, the algorithm tries to artificially increase the sensitivities of compliance in the preferred zones to deposit more material in those areas. This scaling, however, is proportional to the difference between the current density of an element in the structure being optimized $x_e$ and the density of the corresponding element in the reference structure $x^{ref}_e$. In contrast, in ESM, we employ a simpler strategy and scale the elemental energies according to the rules defined in Table 1, which results in a better stability of the optimization process and yields designs with clearer boundaries between material and void. In our previous work^34^, we have rigorously compared the performance of both, formal gradient-based approach with an analytical compliance constraint and a heuristic ESM using SIMP, and have demonstrated the superiority of the novel ESM approach, which can be used together with gradient-based and nongradient TO methods. In this work, we use ESM in an unchanged form, however, instead of running multiple TO runs for different values of the energy scaling factor *p*, we complement the method by employing an ML-based hyperparameter predictor as described in the next section.

**Table 1:** Elemental energy scaling scheme employed by ESM.

Area	SIMP (min. compliance)	HCA (min. compliance)	HCA (max. crash energy absorption)
Preferred	$\frac{\partial c}{\partial x}:=p\cdot \frac{\partial c}{\partial x}$	$SED:=p\cdot SED$	$IED:=p\cdot IED$
Nonpreferred	$\frac{\partial c}{\partial x}:=(1-p)\cdot \frac{\partial c}{\partial x}$	$SED:=(1-p)\cdot SED$	$IED:=(1-p)\cdot IED$

Depending on the location of the elements w.r.t. the reference design, the energies are scaled up or down using the scaling factor *p*. In case of gradient-based compliance (*c*) minimization problems, this is equivalent to scaling of the compliance sensitivities. For HCA, we scale the Strain Energy Density (SED) for static problems or internal energy density (IED) for nonlinear dynamic crash scenarios.

### Learning hyperparameter predictor

**Figure 3:**
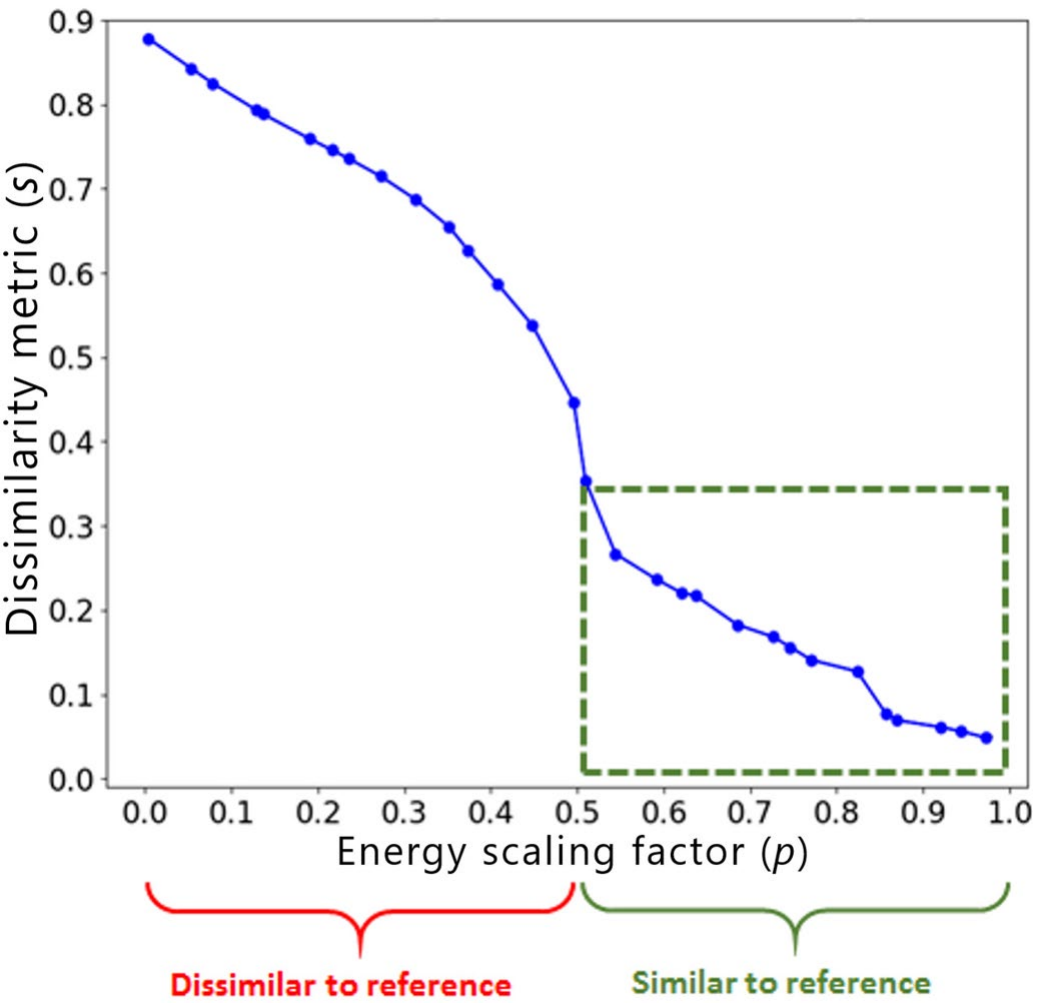
Typical relationship between the dissimilarity metric (*s*) of the topologically-optimized design w.r.t. reference structure and the applied energy scaling factor (*p*)^48^. The green dashed box shows the part of the curve to be predicted by the hyperparameter predictor. Please note that for $p\in (0.5,1.0]$ only designs similar to the reference are generated while running TO for $p=0.5$ is equivalent to standard TO without similarity constraints.

This section proposes a novel approach for building hyperparameter predictors for TO problems, which we describe in the context of similarity-driven TO based on ESM. The process consists of three steps. Firstly, we propose a fully automatic method for generating training data based on a standard 2D TO test case. Secondly, taking into account the sensitivity analysis described in our previous publication^34^, we extract the features that are the key determinants of the relationship between the dissimilarity metric of the topologically-optimized design (*s*) and the energy scaling value (*p*). A typical *s*(*p*) function is illustrated in Fig. 3. Finally, using the automatically generated data and the proposed feature extractors, we build a regression model for predicting attainable similarity levels for different values of the hyperparameter *p*, which is the crucial component of the end-to-end ESM.

#### Training set generation

Since in most industrial applications designs similar to a certain reference structure are of interest, as illustrated in Fig. 3, we limit the analysis presented in this paper exclusively to the energy scaling values greater than or equal to 0.5. However, in fact, also dissimilar structures could be generated for $p\in \left( 0.5, 1.0\right]$ by applying a transformation $x^{ref}_e:=1-x^{ref}_e$ to each of the elements *e* of the reference structure.

**Figure 4:**
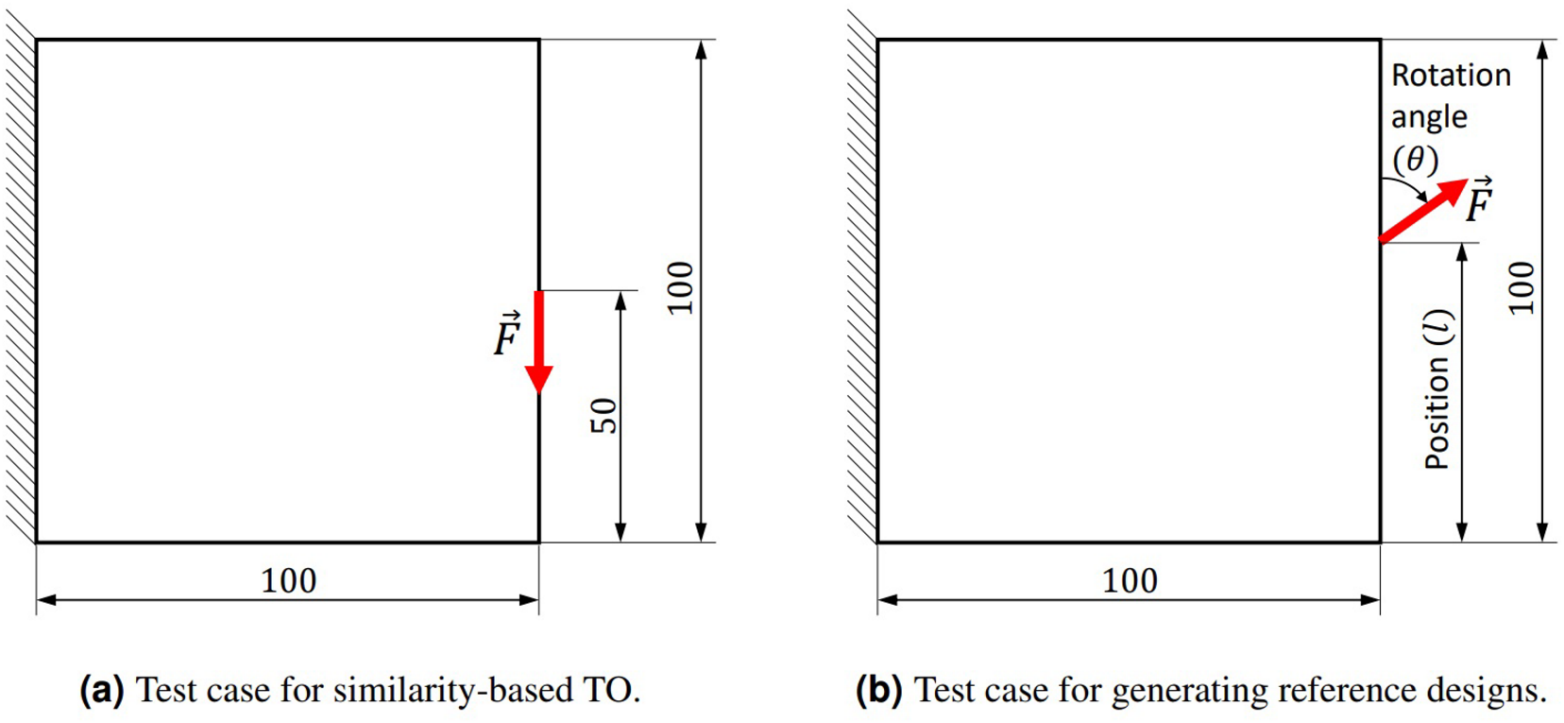
Design domain and boundary conditions of the test case (**a**) used in similarity-based TO for different values of the energy scaling factor *p* to generate training data. The corresponding reference structures are generated using standard TO^49^ based on a design space and boundary conditions parameterized as shown in (**b**). In both cases, a force of magnitude $|\vec {F}|=1\,\text {(N)}$ is applied. The dimensions are defined in (mm).

In this paper, we propose to generate the training data for the regression model using a standard, linear elastic cantilever beam test case with random variations of loading conditions and the volume constraint, which is a standard benchmark problem in TO. The design domain (Fig. 4) consists of $100 \times 100$ square finite elements, with all degrees of freedom on the left edge of the structure being fixed. The material properties of the used mechanical model are defined in Table 2. As we show later, despite the simplicity of this optimization scenario, which helps to reduce the computational effort for the data generation process, the hyperparameter predictors obtained based on such data generalize to both 3D linear elastic and nonlinear dynamic TO problems.

**Table 2:** Description of the mechanical parameters used in the simulation model as well as the optimization hyperparameters of the SIMP algorithm used in the automatic training set generation.

Parameter	Force	Mesh size	Element type	Poisson’s ratio	Young’s modulus	SIMP power	Filter radius
Value	1 (N)	$100\times 100$	4-node shell	0.3	1 (MPa)	3	1.5

To achieve high diversity of the training set, we generate 600 test case-reference pairs, where each pair consists of the test case depicted in Fig. 4a and a reference design generated by running minimal-compliance SIMP TO^49^ for a random angle $\theta$, position *l* of the load along the right edge of the design domain (Fig. 4b), and a volume fraction $f_r\in \left[ 0.2, 0.8\right]$. Based on the provided test case-reference pair, a set of 12 similarity-based TO runs is performed using ESM in SIMP, for energy scaling factor *p* values distributed uniformly in range $\left[ 0.5, 0.98\right]$. Here, also a compliance minimization problem for a randomly chosen target volume fraction $f_t\in \left[ 0.2, 0.8\right]$ is performed. The randomization of the sampling parameters $\theta$, *l*, $f_r$, and $f_t$ is realized using an Optimized Latin Hypercube Sampling (OLHS)^29^, which helps to improve the distribution of the data in the training set.

**Figure 5:**
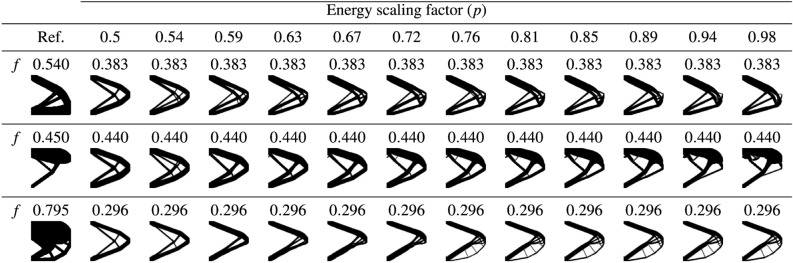
Selected designs from the training set. Each of the rows in the table presents designs obtained via similarity-based TO for a different reference structure, shown in the first column. Above each structure, we specify its volume fraction (*f*). Please note that the designs presented in the second column, i.e., for $p=0.5$, correspond to a standard TO result without any similarity constraints.

As a result of the sampling process involving 7800 TO runs, a set of 7200 training samples is generated, where each of them consists of a structure optimized using ESM in SIMP and the corresponding reference structure. Figure 5 shows selected samples of the resulting dataset.

#### Feature extraction

As indicated in our prior publication^34^ based on the performed sensitivity analysis, the features which have the strongest impact on the relationship between the dissimilarity metric of the optimized design *s* and the energy scaling factor *p* are:

- Target volume fraction of the structure being optimized ($f_t$).
- Reference design’s volume fraction ($f_r$). Intuitively, if the difference between $f_t$ and $f_r$ is big (small) for $p>0.5$, the attainable levels of similarity are low (high) since the intersection area between the optimized and the reference design has to remain small (large).
- Dissimilarity metric of a standard TO optimization result (without similarity constraints) w.r.t. the reference design ($s_r$). The higher the dissimilarity metric the more difficult it is to reduce it even for very high values of *p*, which results in a more “steep” shape of the curve shown in Fig. 3 for $p>0.5$.

**Figure 6:**
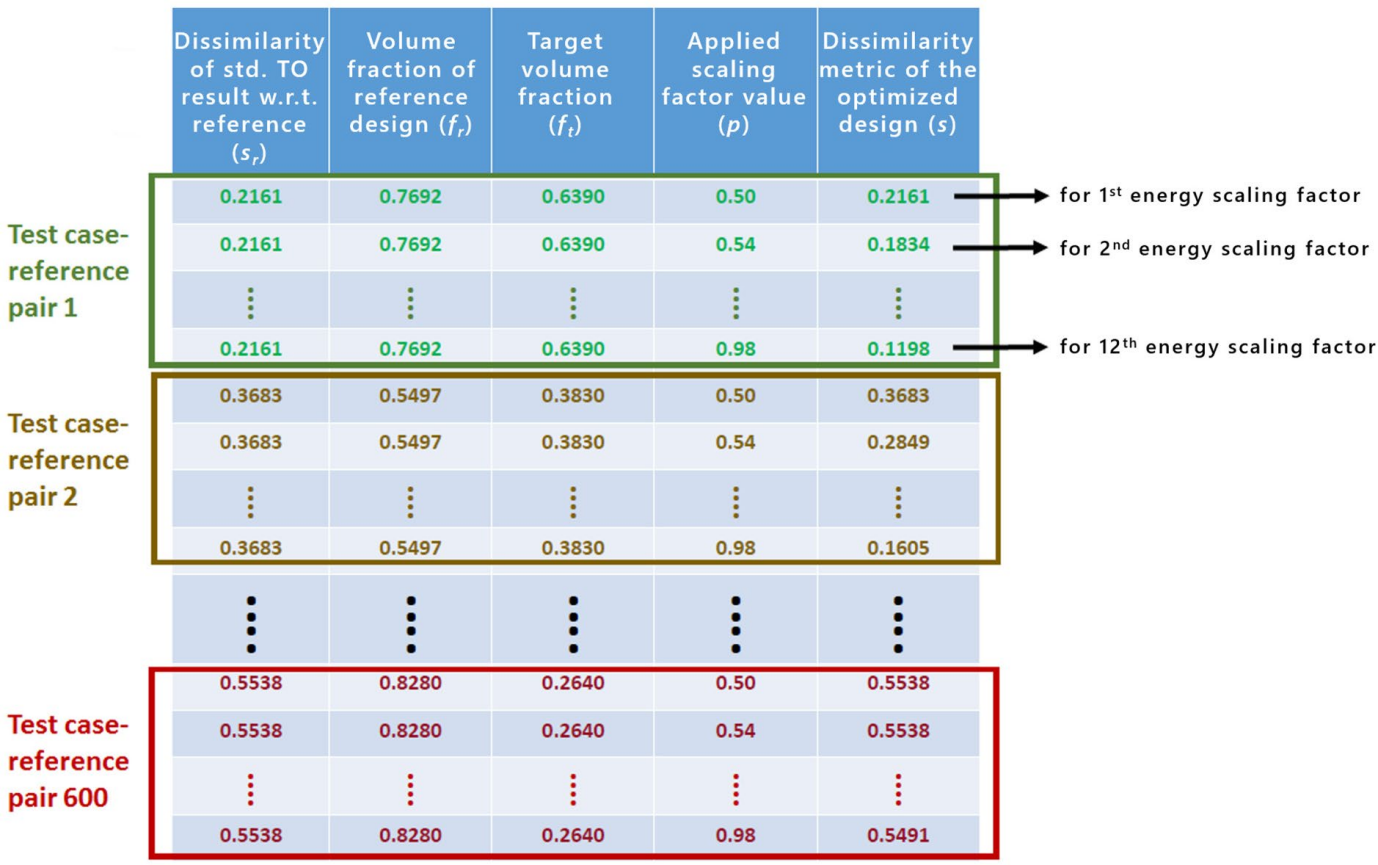
Result of the feature extraction process for selected samples in the automatically generated dataset of structures.

In this paper, we use the features defined above to extract the relevant information from the dataset generated as described in the previous section. In addition, since we are mainly interested in predicting the dissimilarity metric for different values of the scaling factor *p*, it is also used as a feature in the regression model. For a given case, represented in the dataset by a single test case-reference pair, the features $f_t$, $f_r$, and $s_r$ remain constant. In order to compute $s_r$, a single standard TO without similarity constraints has to be carried out. Figure 6 shows a result of the feature extraction process for selected samples of the generated dataset of structures.

#### Regression model

For simplicity, and to assure high generalization of the regression models despite training exclusively on data generated based on 2D linear elastic test cases, we restrict the analysis presented in this paper to polynomial regression models of the input features $f_t$, $f_r$, $s_r$, and *p*, which are used to predict the output property—dissimilarity metric *s* of the design resulting from the ESM-based TO process w.r.t. the reference. Polynomial regression models are easier to interpret and much faster to train and evaluate than more complex models like artificial neural networks, as well. For a given degree *d* of the polynomial model, all polynomial combinations of the input features of degree less than or equal to *d* are used. For instance, for $d=2$, the resulting model consists of 15 polynomial features and takes the form $s \approx f(f_t, f_r, s_r, p) = a_0 + a_1 f_t + a_2 f_r + a_3 s_r + a_4 p + a_5 f_t f_r + a_6 f_t s_r + a_7 f_t p + a_8 f_r s_r + a_9 f_r p + a_{10} s_r p + a_{11} {f_t}^2 + a_{12} {f_r}^2 + a_{13} {s_r}^2 + a_{14} p^2$.

We evaluate the performance of polynomial regression models for $d \in \left\{ 1,2,3,4,5,6,7,8\right\}$ using the well-known *k*-fold cross validation technique with $k=10$, based on a subset of 6480 data samples corresponding to 540 test case-reference pairs, which constitutes $90\%$ of the original dataset. The remaining $10\%$ of the dataset (60 test case-reference pairs) is kept aside as a test set and is used neither for training nor validation of the models. Please note that we always operate on chunks of data samples, where each chunk corresponds to a set of similarity-based TO runs for 12 different values of parameter *p* and the same reference structure. As a result, in model testing, we can predict the entire *s*(*p*) curve based exclusively on the unseen data points.

**Figure 7:**
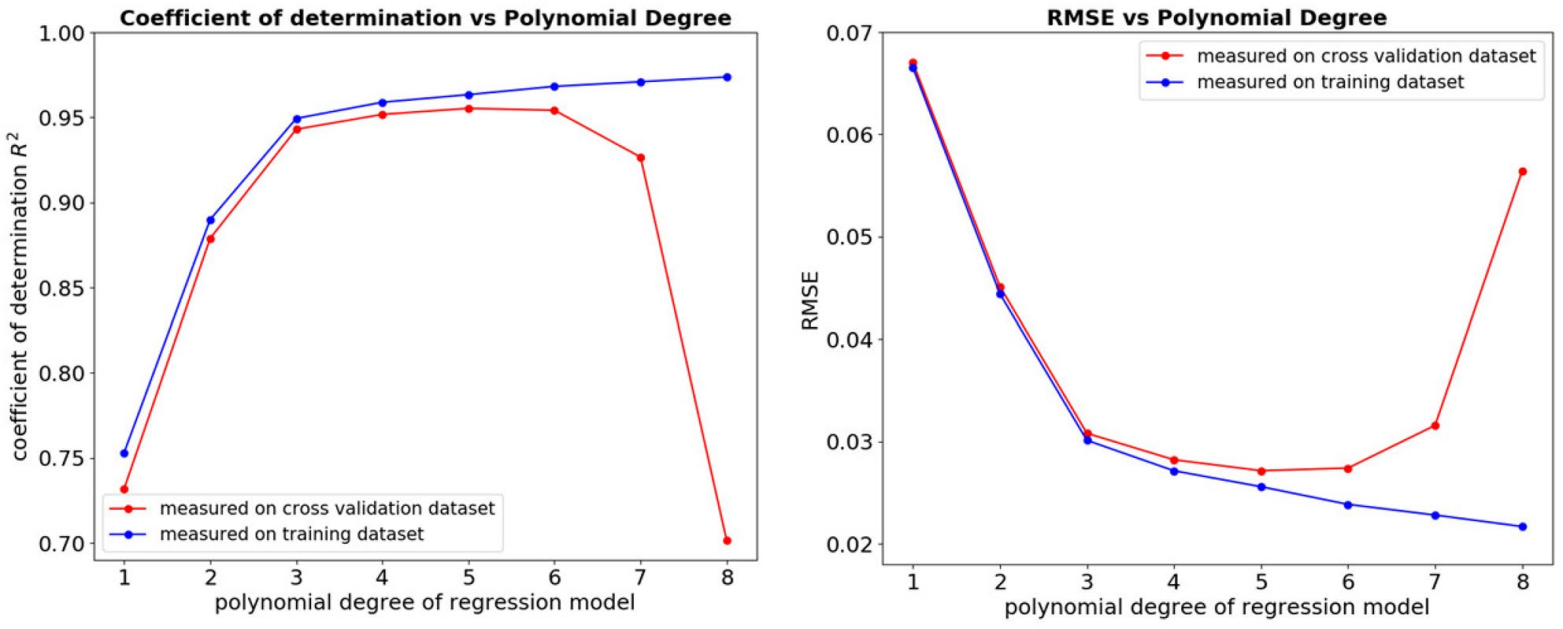
Average values of $R^2$ and RMSE calculated based on training and validation set, for polynomial regression models of different degrees.

Figure 7 presents the coefficient of determination ($R^2$) and the Root Mean Square Error (*RMSE*) values computed on training and validation set for models of different degree *d*, averaged over the *k* folds. One can easily note that the accuracy of the model on the training and validation set gradually increases with the degree of the polynomial regression model, but for $d=5$, the model starts to overfit, which is demonstrated by the decreasing performance on the validation set. Hence, for the further analysis, unless stated otherwise, we choose the model of degree 5 as the one giving the best results on the unseen data samples. When evaluated on the separate test set, the model yields $R^2=0.96$ and $RMSE=0.027$, which is comparable to the performance obtained in the cross validation set.

**Figure 8:**
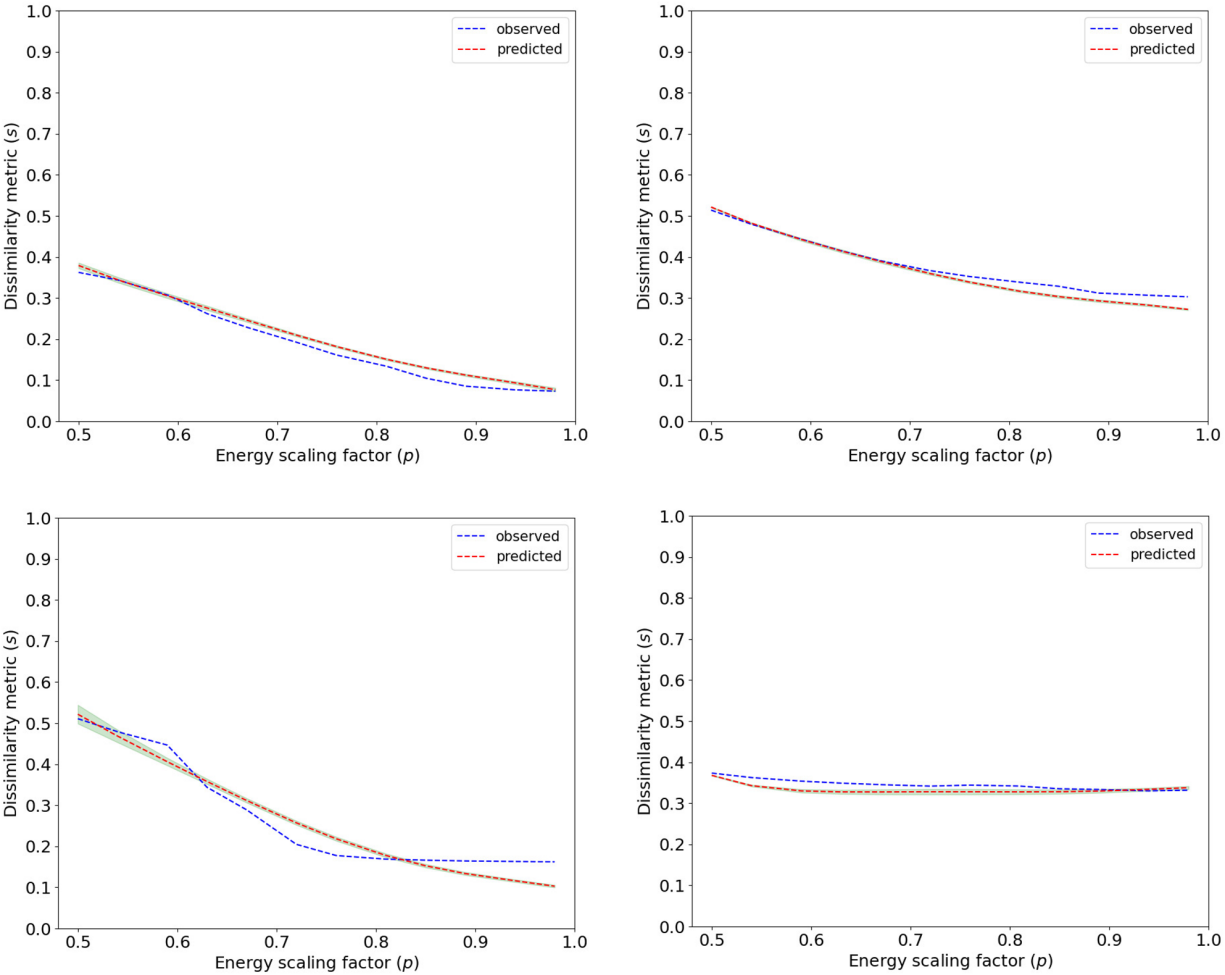
Relationship between the dissimilarity metric *s* and the energy scaling value *p* for randomly selected test case-reference pairs from the test set, with the corresponding predictions of the best polynomial regression model. The green, transparent areas in the plots correspond to a 99% confidence interval, estimated based on 10-fold cross validation using polynomial regression models of degree 5.

To better illustrate predictive capabilities of the model, in Fig. 8, we show randomly selected *s*(*p*) curves from the test set and the corresponding inferences of the obtained model. The model is able to accurately predict different types of *s*(*p*) relationships based on basic characteristics of the standard TO result, expressed by its volume fraction $f_t$ as well as dissimilarity to the reference design $s_r$, and the volume fraction of the reference structure $f_r$. By predicting the entire *s*(*p*) curve, we provide the designer with a possibility to understand the influence of parameter *p* on the attainable dissimilarity levels *s*, and to select a suitable value of *p*. Please note that we predict the *s*(*p*) characteristic based on a single TO run, which, in comparison to the previously used sampling-based approach^33,34^ results in 12 times lower computation time for the sampling resolution used in our experiments.

## Evaluation of the regression model

In this section, a sensitivity analysis of the obtained polynomial regression model is presented to investigate the impact of different input features. Please note that we consider only the predictions of the model and do not compare the results with the ground truth, which is not possible due to the unavailability of the data points in certain regions of the parameter space of the model.

**Figure 9:**
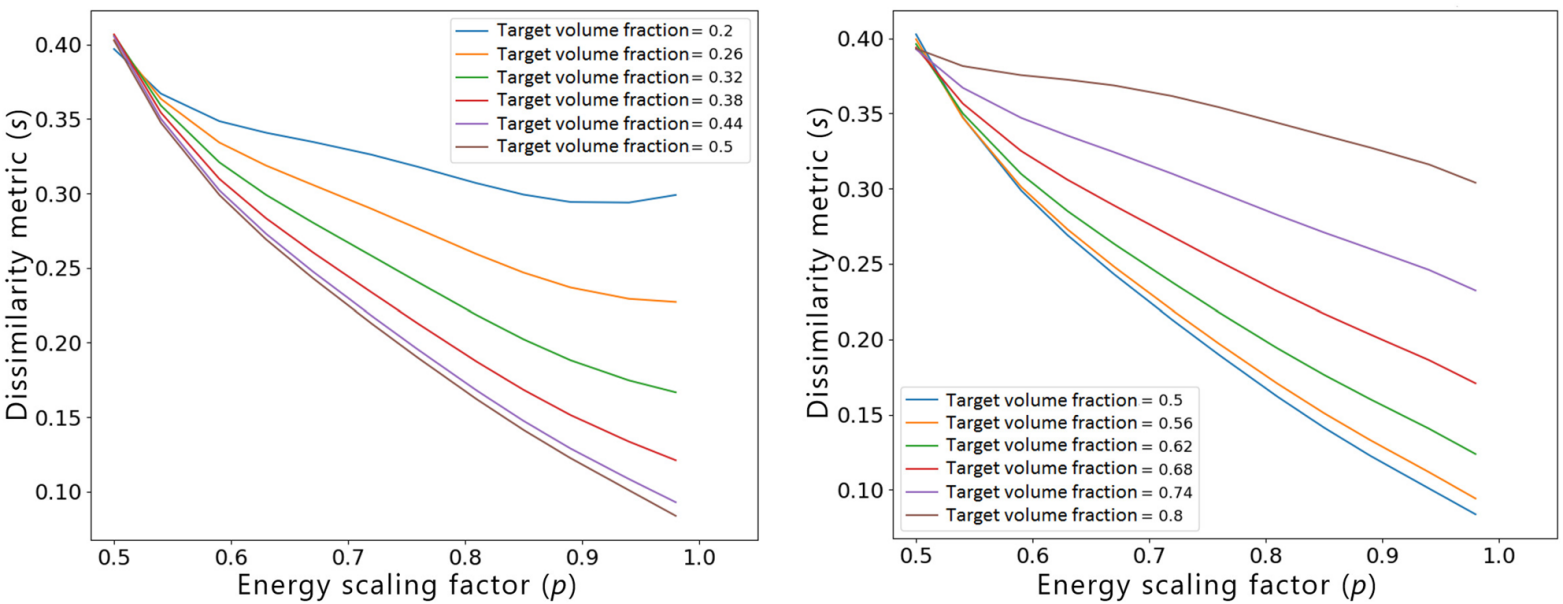
Influence of the target volume fraction $f_t$ on the relationship between the dissimilarity metric *s* and the energy scaling factor *p*. The presented curves were generated based on a polynomial regression model of degree 5, for $s_r=0.4$ and $f_r=0.5$.

First of all, we investigate the influence of the target volume fraction $f_t$ on the *s*(*p*) model. Figure 9 shows a result of such an analysis for fixed values of $s_r=0.4$ and $f_r=0.5$. The behavior of the model is consistent with the intuition since the attainable dissimilarity levels are high for all values of *p* if $\left| f_t-f_r\right|$ is large, because the overlap between the optimized design and the reference structure for such cases is small.

**Figure 10:**
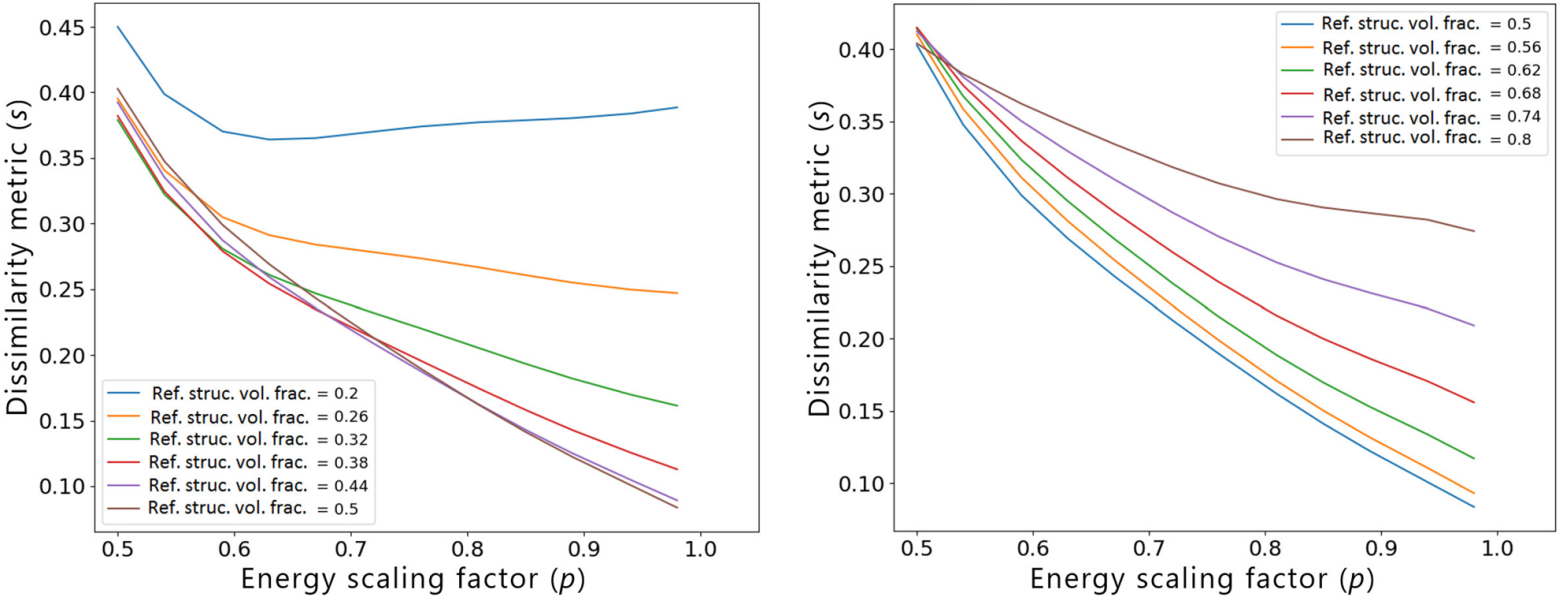
Influence of the volume fraction of the reference design $f_r$ on the relationship between the dissimilarity metric *s* and the energy scaling factor *p*. The presented curves were generated based on a polynomial regression model of degree 5, for $s_r=0.4$ and $f_t=0.5$.

Secondly, Fig. 10 presents a result of the sensitivity analysis of *s*(*p*) model w.r.t. the volume fraction of the reference design $f_r$, for fixed values of $s_r=0.4$ and $f_t=0.5$. Again, for high values of $\left| f_t-f_r\right|$, the dissimilarity metric of the optimized design can be reduced only to a limited extent irrespective of the value of *p*, which is an expected outcome.

**Figure 11:**
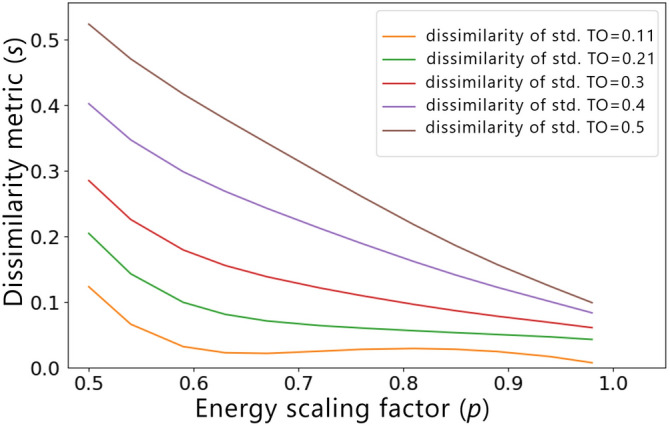
Influence of the dissimilarity of the standard TO result w.r.t. reference $s_r$ on the relationship between the dissimilarity metric *s* and the energy scaling factor *p*. The presented curves were generated based on a polynomial regression model of degree 5, for $f_r=0.5$ and $f_t=0.5$.

Finally, in Fig. 11 we analyze the influence of the dissimilarity metric w.r.t. the reference design computed for a standard TO result without any similarity constraint, for constant values of $f_r=0.5$ and $f_t=0.5$. For cases where the standard TO result is already similar to the reference structure, ESM can influence the material distribution to a very little extent, irrespective of the applied value of the energy scaling factor *p*. As a result, relatively “flat” *s*(*p*) curves are obtained for these cases. The higher the dissimilarity of the standard TO result, the greater the influence of *p* on the final value of *s*, which is in good agreement with a common sense.

In all of the investigated scenarios, a non-monotonic behavior of the polynomial regression models at or close to the lower bounds of the feature intervals, i.e., for $f_t=0.2$ in Fig. 9, $f_r=0.2$ in Fig. 10, and $s_r=0.11$ in Fig. 11, can be observed. Since when increasing the value of the energy scaling parameter *p*, the dissimilarity metric of the optimized design *s* should decrease, the non-monotonicity is most probably associated with the well-known Runge’s phenomenon^50^, which manifests itself through high oscillations of higher-degree polynomial regression functions at the ends of the intervals, especially when using evenly spaced sampling points as the ones generated via OLHS. To improve the accuracy of the model close to the variables’ interval bounds, one could consider increasing the number of sampling points in these areas as well as modifying the distribution of the sampling points. Alternatively, other regression models could be used to mitigate this phenomenon, as well.

## Real-world applications

In this section, we evaluate the capability of the end-to-end similarity-based TO framework to address real-world problems beyond the standard linear elastic cases which were used in this work for learning the predictor of the hyperparameter *p*. We consider two scenarios to test different challenges met in industrial, multidisciplinary TO problems. In the first scenario, we introduce a dynamic 2D crash TO problem, to examine the influence of nonlinearities of the objective function on the predictive capabilities of the proposed hyperparameter model. In the second test case, we evaluate the ability of the model to generalize to complex 3D scenarios by addressing a large-scale car hood frame optimization problem subject to static loading conditions.

### Test cases

Below, the two real-world TO test cases are described in detail.

#### Nonlinear dynamic 2D crash problem

**Figure 12:**
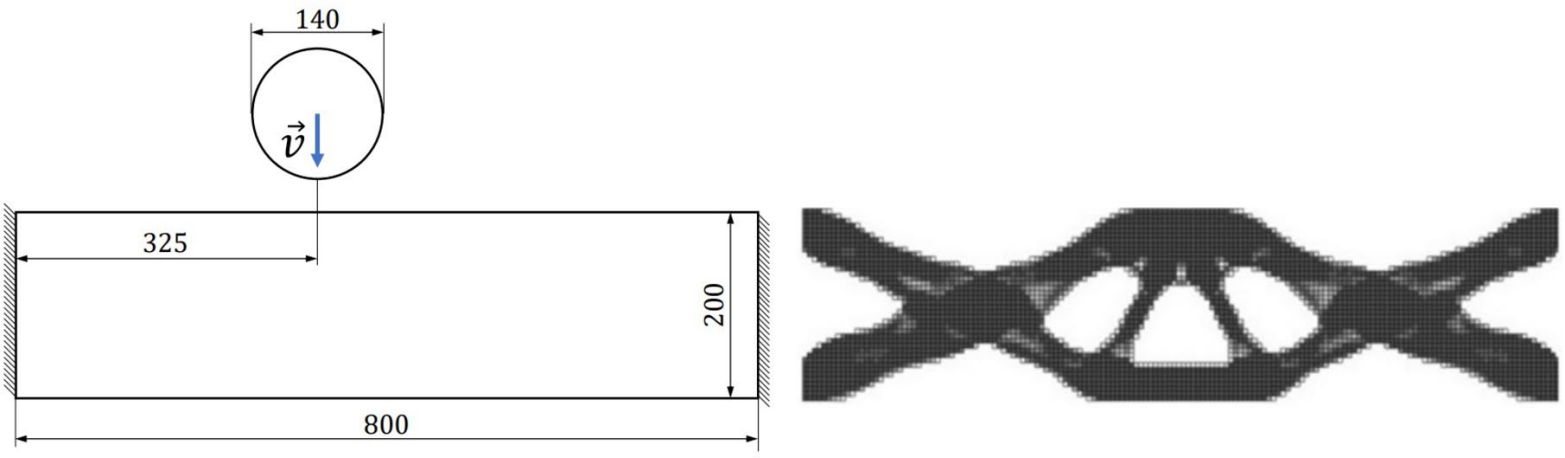
2D crash test case (left) and the reference structure used in similarity-based TO (right). The pole impacts the beam with speed $|\vec {v}|=20\,\text {(m/s)}$. The dimensions are defined in (mm). The out-of-plane thickness of the beam is 5 (mm).

In order to analyze the robustness of the proposed end-to-end framework w.r.t. the nonlinear characteristics of the simulation model, we propose to use a 2D crash scenario as depicted in Fig.  12, which we utilized also in the experiments in our prior publications^33,34^. A rectangular beam is fixed at the right and left edge and impacted by a cylindrical pole from the top. To solve the crash TO problem, we use the state-of-the-art HCA method, which targets homogenization of the internal energy density all over the structure^14^. Consequently, the method implicitly maximizes the energy absorption by the structure while constraining its volume to a predefined value, usually expressed by a volume fraction. Table 3 summarizes the optimization and simulation parameters of the utilized simulation model.

**Table 3:** Parameters used in simulation and optimization of the 2D crash test case.

Parameter	Value
Pole mesh	22 elements
Pole element type	4-node shell
Pole mass density	$5.0\times 10^{-6}$ $(\text {ton/mm}^{3})$
Beam mesh	$160\times 40\times 1$
Beam element type	8-node solid
Beam mass density	$2.7\times 10^{-9}$ $(\text {ton/mm}^{3})$
Beam Young’s modulus *E*	$7.0\times 10^{4}$ (MPa)
Beam Poisson’s ratio $\nu$	0.33
Beam yield strength $\sigma _y$	241 (MPa)
Beam tangent modulus $E_{tan}$	70 (MPa)
LS-Dyna material card (beam)	*MAT_PIECEWISE_LINEAR_PLASTICITY
LS-Dyna material card (pole)	*MAT_RIGID
Volume fraction	0.5
Filter radius	5.5 (mm)

#### 3D car hood optimization

**Figure 13:**
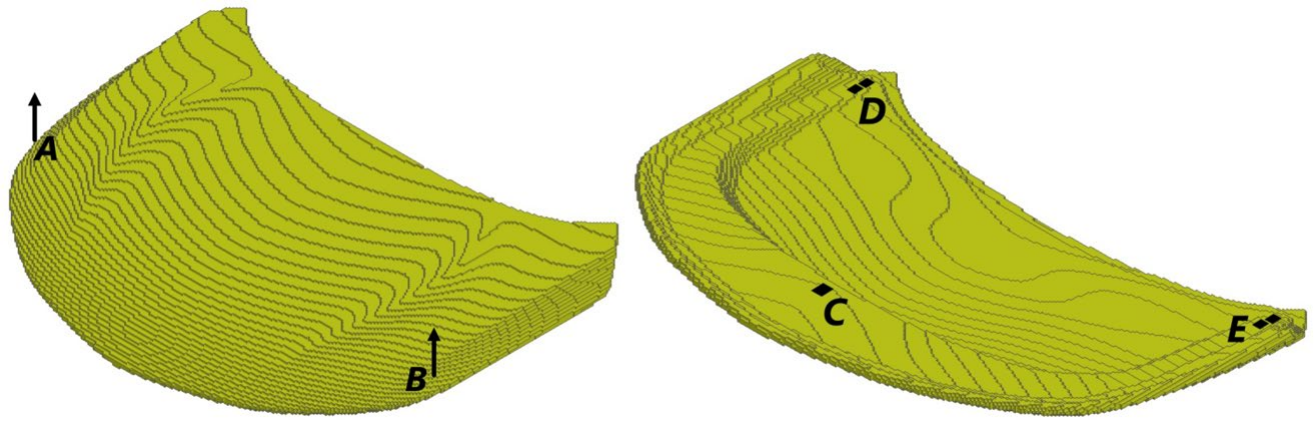
Industrial 3D car hood frame test case used in similarity-based TO. Isometric view from the top and bottom. At points A and B, prescribed displacements of 2 (mm) are defined. All degrees of freedom of the finite element model’s nodes in areas C, D, E are fixed. Moreover, a distributed gravity load is applied to the entire model. In TO, a symmetry condition on elemental densities w.r.t. the light blue plane is imposed.

**Figure 14:**
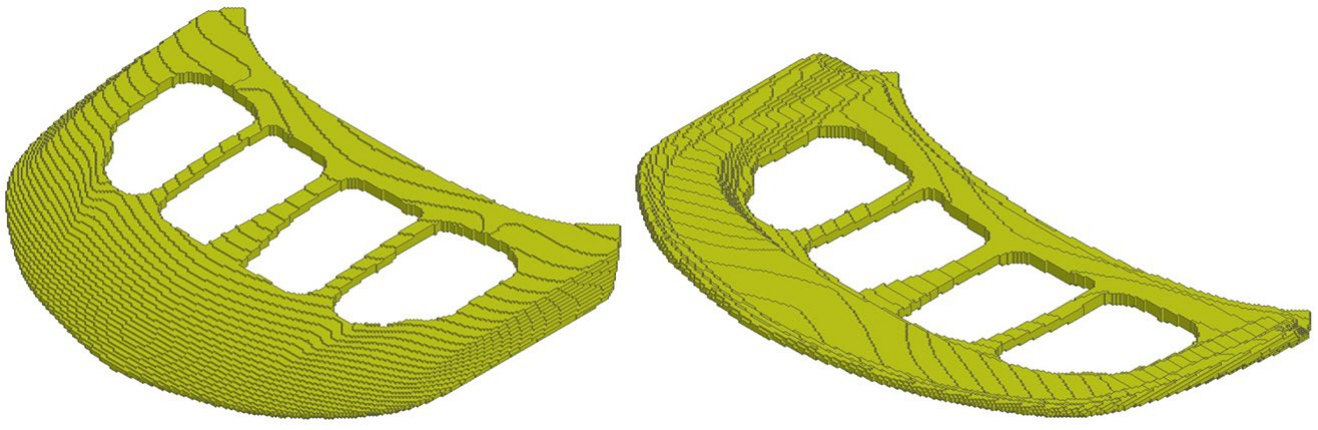
Reference used in the similarity-based TO of the industrial 3D car hood frame. Isometric view from the top and bottom. The model was created by designers of Honda Development & Manufacturing of America and represents the preferred locations of material according to the limitations of the manufacturing and assembly process.

We evaluate the generalization of the proposed end-to-end framework to complex 3D problems based on a hood frame optimization scenario considered already in our prior publication^34^. Optimization of car hood frames is a prominent example of a multidisciplinary design problem, where multiple static and crash load cases have to be taken into account^28,51^ along with criteria related to durability, cost, manufacturability, and the assembly process. The test case considered here is presented in Fig. 13 and the corresponding simulation and optimization parameters for this problem are listed in Table 4. Without loss of generality, we consider only a single static load case, which represents loading of the structure according to the aerodynamic forces acting on the hood skin. However, in HCA, which we use also for TO of the car hood frame, both static and crash load cases can be considered concurrently^21^, and it is also possible to use ESM in such scenarios. In this work, we encapsulate the criteria related to other disciplines, in particular the limitations of the manufacturing and assembly process, by defining a reference as depicted in Fig. 14.

**Table 4:** Parameters used in simulation and optimization of the industrial 3D car hood frame test case.

Parameter	Value
Number of elements	195, 830
Element type	8-node solid
Prescribed displacement	2 (mm)
Mass density $\rho$	$2.7\times 10^{-9}$ $(\text {ton/mm}^{3})$
Young’s modulus *E*	$6.7\times 10^{4}$ (MPa)
Poisson’s ratio $\nu$	0.32
Volume fraction	0.35
HCA filter radius	10.5 (mm)
Symmetry constraint	as shown in Fig. 13

### Results

In this section, we apply the end-to-end similarity-driven TO approach based on the hyperparameter predictor to the test cases described above and discuss the obtained results. The primary goal of this section is to investigate the generalization capabilities of the hyperparameter model on unseen data corresponding to challenging real-world problems.

#### Nonlinear dynamic 2D crash problem

**Figure 15:**
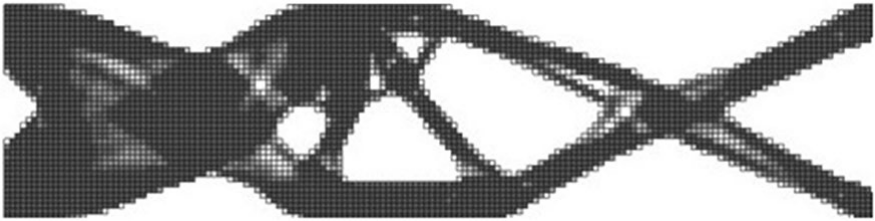
Design obtained using standard HCA algorithm without similarity constraints for the 2D crash TO problem.

First of all, we consider the 2D nonlinear dynamic crash scenario depicted in Fig. 12 and summarized in Table 3. To calculate the input features for predicting the *s*(*p*) relationship in the proposed end-to-end framework, at first, one has to perform standard TO to extract the relevant features. The result of such a TO run is illustrated in Fig. 15 and the corresponding features are given in Table 5.

**Table 5:** Input features for the hyperparameter predictor, calculated for the 2D crash TO problem.

Input feature	Value
Volume fraction of the reference design $(f_r)$	0.50
Dissimilarity of reference w.r.t. standard TO design $(s_r)$	0.27
Target volume fraction $(f_t)$	0.50
Energy scaling factor (*p*)	$p\in [0.5, 0.98]$

**Figure 16:**
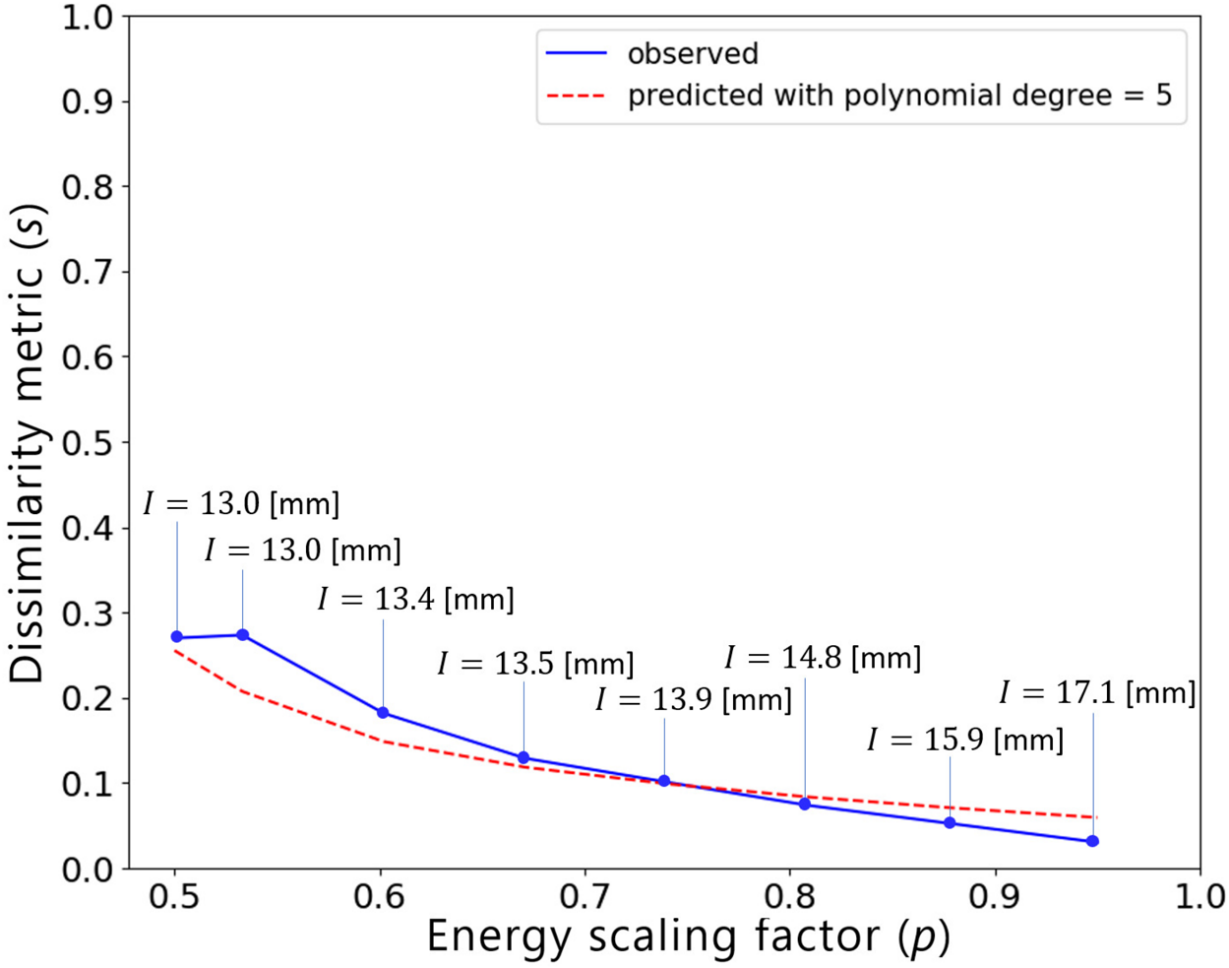
Relationship between the dissimilarity metric *s* and the energy scaling value *p* predicted by the proposed hyperparameter model (dashed red curve) and the corresponding curve obtained via performing multiple similarity-based TO runs^34^ (solid blue curve) for the 2D dynamic crash TO problem, together with the corresponding structural performance metric, i.e., intrusion (*I*) of the pole into the structure.

**Figure 17:**
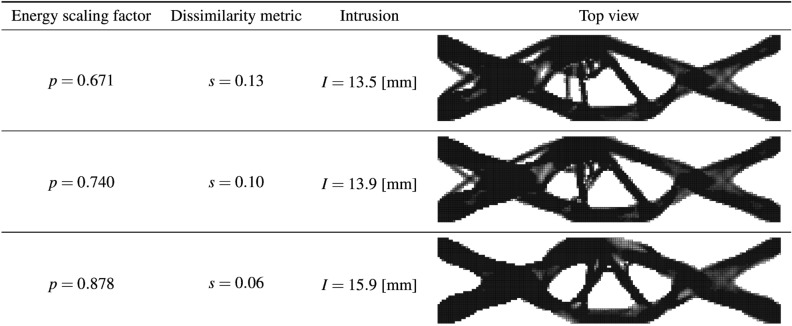
Designs optimized using the end-to-end ESM TO framework for the 2D crash test case and three different similarity levels.

Given the input features, the model trained based on linear elastic SIMP TO results is used to predict the *s*(*p*) relationship. Figure 16 presents the predicted curve together with the actual dissimilarity metric values (ground truth) computed based on similarity-driven TO results for the 2D crash scenario, which are described in our prior publications^33,34^. Based on the obtained results, one can easily note that the hyperparameter model generalizes well to a scenario where both a different TO method, HCA, is used, and the underlying physical problem involves nonlinearities. In addition, using the predicted curve, the designer can easily inspect the attainable levels of dissimilarity metric *s* and estimate the value of *p* needed to reach a specific value of *s*. Figure 17 presents structures optimized using the end-to-end similarity-based TO framework for three selected similarity levels.

#### 3D car hood optimization

**Figure 18:**
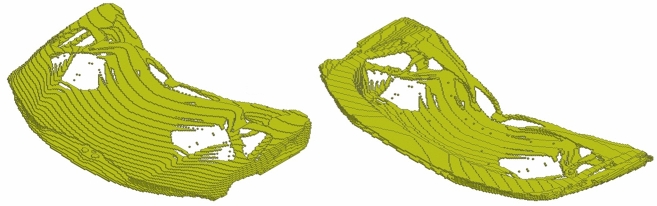
Design obtained using standard HCA algorithm without similarity constraints for the 3D hood frame TO problem. Isometric view from the top and bottom.

**Figure 19:**
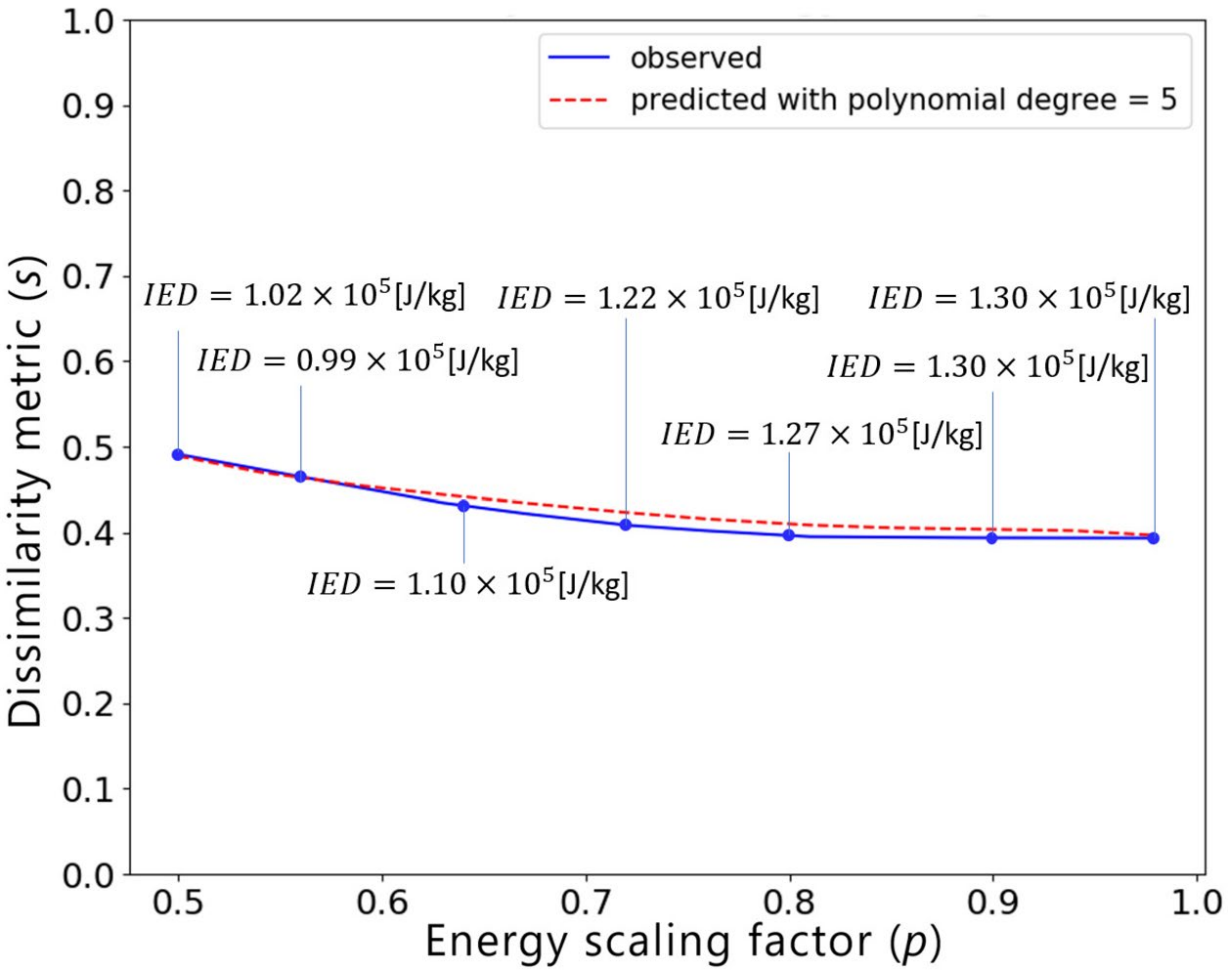
Relationship between the dissimilarity metric *s* and the energy scaling value *p* predicted by the proposed hyperparameter model (dashed red curve) and the corresponding curve obtained via performing multiple similarity-based TO runs^34^ (solid blue curve) for the 3D hood frame TO problem, together with the corresponding structural performance metric, i.e., Internal Energy Density (IED).

**Figure 20:**
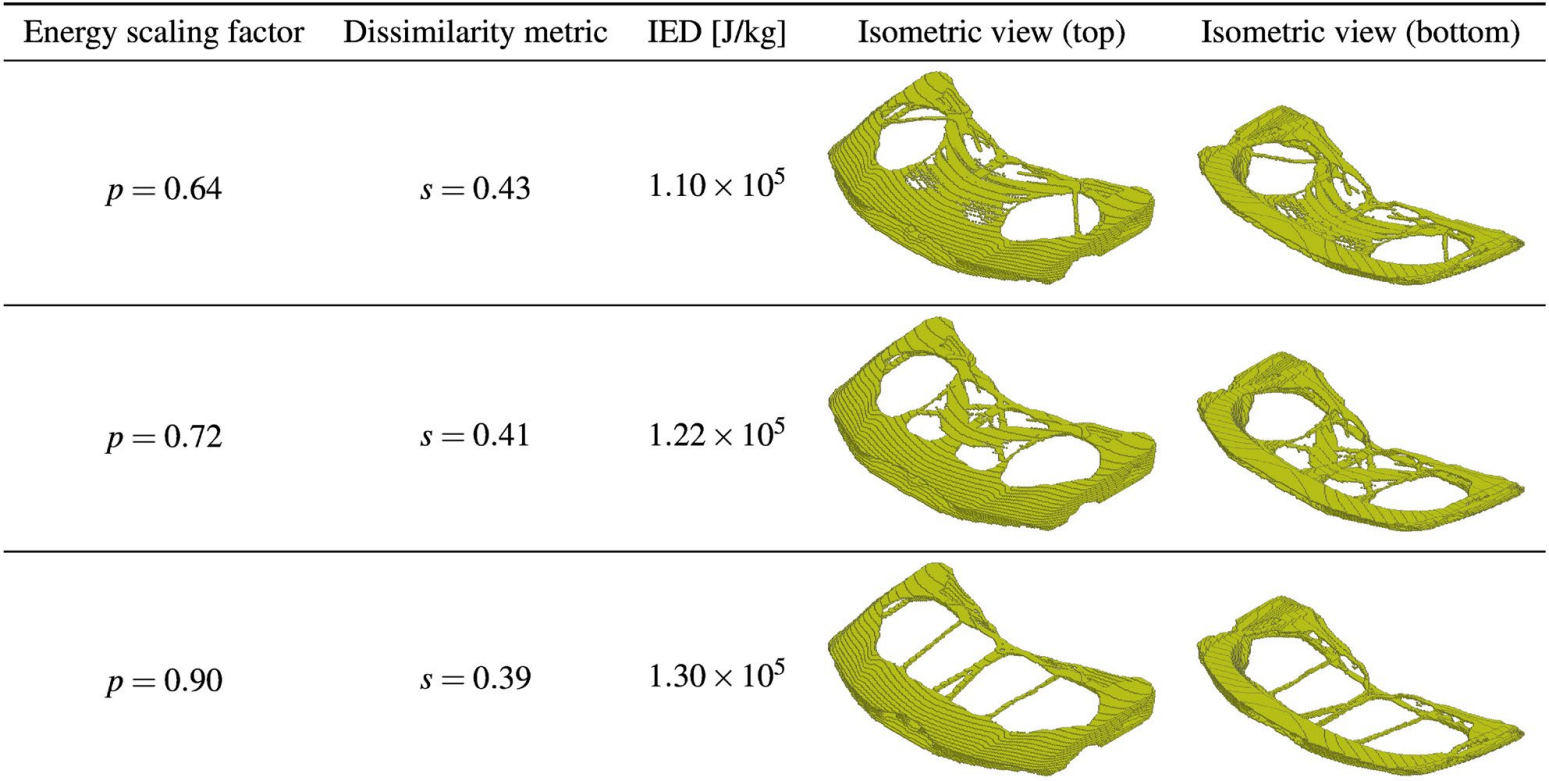
Selected hood frame designs optimized using the end-to-end ESM TO framework for different similarity levels.

In this section, we present the application of the end-to-end similarity-based TO framework to the industrial 3D car hood optimization scenario depicted in Fig.  13. Figure 18 shows a standard TO result (without similarity constraints) for the minimal compliance optimization using HCA. Based on the resulting structure as well as the reference design (Fig.  14), the input features for predicting *s*(*p*) curve are computed and are given in Table 6. The predicted values of the dissimilarity metric *s* for different levels of *p* are presented in Fig.  19 along with the actual dissimilarity metrics (ground truth) computed after running similarity-based TO. The corresponding topologically-optimized structures are described in our past works^34,48^. Surprisingly, also in the case of a complex, large-scale 3D TO problem, the predictions of the hyperparameter model trained on academic linear elastic test cases are in a good agreement with the underlying ground truth data. Hence, the model allows for an accurate estimation of the required energy scaling value to reach the desired dissimilarity metric *s*. As can be seen in Fig.  19, for the test case considered here, the dissimilarity metric *s* can be varied to a very small extent by adjusting the parameter *p*, which is most probably caused by a high difference between the target volume fraction ($f_t=0.35$) and the volume fraction of the reference design ($f_r=0.76$). Finally, in Fig. 20, we show designs optimized using the proposed end-to-end framework for three selected values of the energy scaling factor *p*. Please note that in case of the structures obtained for $p=0.72$ and $p=0.90$, the dissimilarity metric changes only slightly, but both designs are topologically very different, which might be counter-intuitive for a human designer. In fact, this is an expected result when using the dissimilarity metric (1) evaluating only the volumetric difference between the structures, which might be almost the same in both cases. The metric used in the paper has an easy physical interpretation—an increase of the dissimilarity metric by 0.01 corresponds approximately to an increase of the volume of the non-overlapping parts of two structures by 1% of the design space volume. In order to quantify topological or other intuitively relevant geometric differences, an interesting extension of the framework presented in this work might be to use alternative dissimilarity metrics, where especially the ones based on deep learning approaches^28,52^ could be promising due to their capability of learning to extract features relevant from the standpoint of the engineering development process^28,53,54^.

**Table 6:** Input features for the hyperparameter predictor, calculated for the 3D hood frame TO problem.

Input feature	Value
Volume fraction of the reference design $(f_r)$	0.76
Dissimilarity of reference w.r.t. standard TO design $(s_r)$	0.49
Target volume fraction $(f_t)$	0.35
Energy scaling factor (*p*)	$p\in [0.5, 0.98]$

All in all, we were able to efficiently solve an industrial-scale multidisciplinary TO problem, where the nonstructural criteria related to costs and the limitations of the manufacturing and assembly process were expressed by defining a reference which incorporates specific engineering know-how. The high efficiency of the algorithm results from the fact that the increase of computational costs due to the utilization of ESM in HCA or SIMP is negligible, since using ESM involves only a simple scaling of elemental energies^33,34^. The generality of the method allows also for an easy integration of criteria related to other physical simulations, e.g., crash. Hence, incorporation of additional load cases into this problem should be straightforward and feasible from the standpoint of computational costs, even on regular workstations used in industry. As such, our end-to-end similarity-based TO framework can be easily applied to other large-scale industrial multidisciplinary problems, where utilization of TO in early design phases plays a critical role due to the high problem complexity resulting from multiple load cases and design criteria that have to be considered concurrently, which often leads to very unintuitive structural concepts. In our opinion, in such cases, the proposed methodology can be a very useful engineering tool able to integrate specific human knowledge and preferences into a mathematical optimization process.

## Conclusion

In this paper, we proposed an end-to-end framework for similarity-based Topology Optimization (TO) based on Energy Scaling Method (ESM)^33,34^. The ESM approach has a generic character and can be used with different types of TO methods to address complex multidisciplinary problems by allowing a designer to encapsulate criteria related to manufacturing, costs, assembly process, or even aesthetics, by specifying a reference design for similarity-based TO. The similarity level of the structure being optimized to the reference design is controlled by adjusting the energy scaling factor, a hyperparameter of ESM. Unlike in the previous approach, in the end-to-end framework, an expensive sampling of different values of the energy scaling factor based on multiple TO runs is no longer needed, and is replaced by a hyperparameter model able to predict the entire relationship between the dissimilarity metric and the energy scaling factor by extracting relevant features from a single TO run. Based on the predicted relationship, the designer is provided with an information about the attainable similarity levels and their sensitivity w.r.t. variations of the energy scaling factor.

We trained the hyperparameter predictor based on a large dataset of designs generated using 2D linear elastic benchmark TO problems, however, it has very good generalization capabilities. We demonstrated it by applying the end-to-end framework to two real-world problems representing typical challenges met in the industrial practice. Based on a 2D dynamic crash TO problem, we showed the generalization capabilities of the proposed hyperparameter predictor, and consequently, the applicability of the end-to-end similarity-based TO framework to problems involving nonlinear and noisy objective functions. Moreover, we studied the ability of the framework to address industrial-scale multidisciplinary TO problems using a hood frame TO test case, where criteria related to manufacturability and costs were incorporated into TO by using a reference design provided by engineers of Honda Development and Manufacturing of America. The hyperparameter predictor generalizes well also to this scenario, which demonstrates good scalability of the method despite training the model based exclusively on much less complex, 2D geometries. Finally, in the sensitivity studies presented in the paper, we analyzed the properties of the hyperparameter predictor to better understand the influence of different input features on the relationship between similarity and the applied scaling factor. The behavior of the model in the sensitivity analyses was consistent with our prior studies and observations.

An interesting direction for the future research would be to integrate into the framework other types of design similarity metrics, where especially the ones based on deep learning could help to improve manufacturability of the optimized designs^52,55^. Moreover, considering multiple reference designs could be very useful in industrial practice in case of both, development of novel structures, and inclusion of specific know-how of companies related to manufacturing process and the associated costs. In such cases, perhaps also more complex, multi-output hyperparameter predictors would be beneficial to estimate the energy scaling factors.

All in all, we strongly believe that the proposed end-to-end similarity-based TO framework, thanks to a very intuitive way of integrating alternative design criteria, which are often very difficult to specify in a closed mathematical form, could play an important role in the future industrial design processes especially when multiple criteria are involved. By integrating the hyperparameter predictor, we were able to considerably reduce the computational costs by eliminating the need for multiple TO runs to determine the required hyperparameter values and explore attainable similarity levels. The future research directions open a range of possibilities to integrate more of the specific design knowledge of companies, contained in large datasets of existing designs, into the similarity-driven design process.

## Data Availability

The datasets as well as simulation models generated and analysed during the current study are available from the corresponding author on reasonable request. The source code of the Energy Scaling Method is available under https://github.com/HRI-EU/Similarity-TO-ESM.
